# Tuberous sclerosis complex exhibits a new renal cystogenic mechanism

**DOI:** 10.14814/phy2.13983

**Published:** 2019-01-23

**Authors:** John J. Bissler, Fahad Zadjali, Dave Bridges, Aristotelis Astrinidis, Sharon Barone, Ying Yao, JeAnna R. Redd, Brian J. Siroky, Yanqing Wang, Joel T. Finley, Michael E. Rusiniak, Heinz Baumann, Kamyar Zahedi, Kenneth W. Gross, Manoocher Soleimani

**Affiliations:** ^1^ Department of Pediatrics University of Tennessee Health Science Center and Le Bonheur Children's Hospital Memphis Tennessee; ^2^ St. Jude Children's Research Hospital Memphis Tennessee; ^3^ Department of Clinical Biochemistry College of Medicine & Health Sciences Sultan Qaboos University Muscat Oman; ^4^ Department of Nutritional Sciences University of Michigan School of Public Health Ann Arbor Michigan; ^5^ Departments of Medicine University of Cincinnati College of Medicine Cincinnati Ohio; ^6^ Center on Genetics of Transport University of Cincinnati College of Medicine Cincinnati Ohio; ^7^ Research Services Veterans Affairs Medical Center Cincinnati Ohio; ^8^ Department of Pediatrics University of Cincinnati College of Medicine Cincinnati Ohio; ^9^ Department of Molecular and Cellular Biology Roswell Park Cancer Institute Buffalo New York

**Keywords:** Intercalated cells, renal cystic disease, renal cystogenesis, Tuberous sclerosis complex

## Abstract

Tuberous sclerosis complex (TSC) is a tumor predisposition syndrome with significant renal cystic and solid tumor disease. While the most common renal tumor in TSC, the angiomyolipoma, exhibits a loss of heterozygosity associated with disease, we have discovered that the renal cystic epithelium is composed of type A intercalated cells that have an intact *Tsc* gene that have been induced to exhibit *Tsc‐*mutant disease phenotype. This mechanism appears to be different than that for ADPKD. The murine models described here closely resemble the human disease and both appear to be mTORC1 inhibitor responsive. The induction signaling driving cystogenesis may be mediated by extracellular vesicle trafficking.

## Introduction

Tuberous sclerosis complex (TSC) is caused by mutations in either the *TSC1* or *TSC2* genes and affects over one million patients world‐wide (Ewalt et al. 1998; Dabora et al. 2001; Rakowski et al. 2006). Over 80% of young patients and all postmortem samples (mean age 30 years (Stillwell et al. 1987)) were found to have renal disease. Renal cystic disease is detected by MRI in ~50% of TSC patients, although the pathogenesis is not well studied. Premature decline of glomerular filtration rate (GFR) occurs in ~40% of patients with TSC (Bissler and Kingswood 2016) and can occur in the absence of overt angiomyolipomata bleeding or interventions and is, at least in part, due to renal cystic disease.

TSC renal cystic disease exhibits five distinct patterns (Bissler 2018; Bissler and Kingswood 2018) and involves the mechanistic target of rapamycin complex 1 (mTORC1) signaling pathway. The mTORC1 signaling pathway integrates intra‐ and extracellular information to regulate cellular metabolism, translation, growth, proliferation, autophagy, and survival and is critical for organogenesis and organ maintenance. The TSC proteins directly regulate mTORC1 activity and influence downstream processes, including renal development, homeostasis, and malignancy. Although the TSC proteins play a pivotal role in cell biology, how their regulation of the mTORC1 pathway is involved in cystogenesis is not known.

The etiology of another common TSC renal lesion, angiomyolipomata, is thought to rely on a somatic mutation mechanism that disables the functional copy of the affected *TSC* locus leading to clonal proliferation of cells lacking TSC‐mediated regulation of the mTORC1 pathway (Lam et al. 2017). There are multiple interactions between mTORC1 signaling and candidate cystogenic mechanisms. Investigation of both *Tsc1‐* or *Tsc2‐*associated renal cystic disease mouse models directly (Onda et al. 1999; Zhou et al. 2009; Armour et al. 2012) or indirectly (Chen et al. 2014) demonstrated that cystogenesis is attributable to specific nephron segments, and all tubular segments have been implicated in *Tsc* cyst formation (Traykova‐Brauch et al. 2008). The identification of the cell of origin for renal cysts is complicated by the tubular epithelial capacity to undergo dedifferentiation during repair/regeneration, and restorative processes that recapitulate renal developmental processes (Dziedzic et al. 2014). Interestingly, all mouse model studies that examined both mTORC1 activity and targeted cells exhibit a mismatch between exuberant cystic phospho‐S6 expression, and the much lower percentage of cells exhibiting loss of Tsc expression (Onda et al. 1999; Zhou et al. 2009; Armour et al. 2012).

Published mouse Tsc models are commonly reported to be born with normal kidneys but cystogenesis progresses with age. One such model has been reported to be associated with a potassium excretion defect (Chen et al. 2014). Early investigation revealed that the majority of renal cysts maintain their *Tsc* locus integrity (Onda et al. 1999; Wilson et al. 2006), as loss of heterozygosity was found in a striking minority of cystic epithelial cells. This is similar to human TSC renal cystic disease, where human cysts continue to express tuberin and hamartin, and this contrasts with a very different mechanism in the formation of angiomyolipomata, which show an inactivating mutation and loss of *Tsc* gene expression (Bonsib et al. 2016). Such a low percentage of loss of heterozygosity is seen also in *PKD1*‐associated autosomal dominant polycystic kidney disease, suggesting that such cystic disease may represent a unique disease mechanism (Brasier and Henske 1997; Badenas et al. 2000).

To build on these initial studies of *Tsc*‐associated renal cystic disease in the mouse model system, we employed two newly developed mouse *Tsc*‐renal cystic disease models, one that disrupts the *Tsc2* gene in renal principal cells, and the other that disrupts the *Tsc1* gene in renal pericytes. These models suggest that, similar to renal development, a tissue induction or reprogramming phenomenon occurs such that cells with an intact Tsc gene adopt *Tsc*‐mutant cell phenotypes, defining a new and distinct mechanism of disease from that reported for angiomyolipomata development. These findings help explain the discrepancies between disease tissue architecture and persistence of TSC protein expression and demonstrate that genetically normal, TSC‐intact cells are induced in large part to participate in cystogenesis.

## Materials and Methods

### Animal procedures

*Ren‐1c‐Cre* mice were generated in the laboratory of K.W. Gross (Glenn et al. 2008). Floxed *Tsc1* mice (stock #005680; (Kwiatkowski et al. 2002)) and Floxed Tsc2 mice (stock #027458) were obtained from The Jackson Laboratory AqpCre mice and “Confetti” mice were also obtained from The Jackson Laboratory. The “Confetti” reporter uses the Brainbow2.1 cassette inserted into the *Rosa26* locus, where it is driven by the *CAGG* strong promoter. The reporter system is activated by excision of a floxed stop sequence by the Cre recombinase. The Brainbow reporter cassette contains two inverted repeats of fluorescent reporter genes: GFP paired with inverted YFP, and RFP paired with inverted CFP. The loxP sites within the construct are in direct and inverted orientations to facilitate loss of the floxed stop module and expression of one of the reporter pairs. The remaining reporter pair can continue to “flip” into the active orientation for one of the two inverted reporters while Cre activity remains present, resulting in bi‐colored cells, and will be locked into one or the other orientation when Cre activity stops (Snippert et al. 2010).

All animal research was done in adherence to the NIH Guide for the Care and Use of Laboratory Animals. These mice were crossed to generate offspring that were heterozygous for the floxed allele, and were either heterozygous or wild‐type at the *Cre* allele. These mice were then intercrossed to generate knockout mice (*Tsc1* ^fl/fl^; *Ren‐1c‐Cre* ^Tg/+^), wild‐type mice (*Tsc1* ^+/+^; *Ren‐1c‐Cre* ^+/+^) and controls for the floxed allele (*Tsc1* ^fl/fl^; *Ren‐1c‐Cre* ^+/+^), *Cre* allele (*Tsc1* ^+/+^; *Ren‐1c‐Cre* ^Tg/+^). A similar strategy was used for the AqpCre lineages. Animals were housed in a 12 h dark‐light cycle according to protocols approved by the University of Tennessee Health Science Center Institutional Animal Care and Use Committee. For rapamycin injections, drug was dissolved in vehicle (5.2% PEG 400/Tween 80) and injected at 3 mg/kg three times per week starting at weaning (21 days of age).

### Perfusions

Three clear plastic tubes were connected by three‐way stopcock to a peristaltic pump. The ends of two tubes were placed in either 0.9% saline or 10% neutral buffered formalin. A 30 &frac12; G needle was attached to the end of the third tube. The formalin tube was filled to the stopcock and the saline tube was allowed to flow from the needle at a rate of 0.01 mL/sec. Prior to perfusion, mice were anesthetized twice, first with isoflurane in a drop until their heartbeat slowed to 1 beat per second, followed by an intraperitoneal injection of 0.8 mL/20 g body weight of Avertin. Once there was no reaction to painful stimuli (e.g., toe pinch), the chest cavity was opened and the diaphragm cut. With the heart exposed, the apex of the left ventrical was punctured with the 30 &frac12; G needle attached to the perfusion liquid tubing and the aorta was cut. Mice were perfused with 0.9% saline, followed by 10% neutral buffered formalin for 10–12 min each. Dissected tissues were stored in formalin overnight, then in 70% ethanol (Pharmaco‐Aaaper) until processing.

### Histology and immunohistochemistry staining

Tissues were processed with the following washes: 30 min in 75% ethanol, 2 × 75 min in 95% ethanol, 3 × 1 h in 100% ethanol, 2 × 30–60 min in Citrosolv (Fisher Scientific) and 2 × 1 h in Paraplast XTRA (Sigma‐Aldrich), with an additional final incubation overnight in Paraplast XTRA under vacuum). Tissues were embedded into blocks using Paraplast XTRA paraffin (Sigma‐Aldrich) in a Thermo Shandon Histocentre 3 paraffin embedding station. For Immunohistochemistry, the kidneys from the knockout mice (*Tsc1* ^fl/fl^; *Ren‐1c‐Cre* ^Tg/+^) and wild‐type mice were prepared in 10% formalin fixed, paraffin embedded. Tissue blocks were cut to 8 μm sections. Immunohistochemical stains were carried out using the The VECTASTAIN^®^ Elite^®^ ABC HRP Kit (Vector Laboratories, Burlingame, CA) with DAB (3,3‐diaminobenzidine) as the chromogen (Vector Laboratories, Burlingame, CA). The antibodies used are presented in Table 1. All the sections were counterstained with Gill2 Hematoxylin (Fisher Scientific, Hampton, NH). In every case, formalin‐fixed tissue was subjected to heat‐induced antigen retrieval. Endogenous peroxidase activity was blocked with 3% H_2_O_2_. Avidin/Biotin Blocking Kit (Vector Laboratories, Burlingame, CA) was used to block all endogenous biotin, biotin receptors, and avidin‐binding sites present in tissues. For all immunohistochemical studies, more that 95% of the slide had the reported pattern of staining.

**Table 1:** List of antibodies used in the study

Antibody	Dilution	Source	Catalog Number
Alexa Fluor Donkey anti‐Goat IgG	1:200	Invitrogen; Eugene, OR	A‐11055
Alexa Fluor Goat anti‐Mouse IgG	1:200	Invitrogen; Eugene, OR	A‐11001
Alexa Fluor Goat anti‐rabbit IgG	1:200	Invitrogen; Eugene, OR	A‐11008
Anti‐Hamartin	1:1000 1:1000	Cell Signaling Technology, Inc. Danvers, MA ProteinTech Rabbit Polyclonal	4906 209988‐I‐AP
Anti‐Na^+^/K^+^ATPase		Dr. Jerry Lingrel, University of Cincinnati	
Anti‐phospho‐P70S6Kinase (Thr389)	1:5000	Cell Signaling Technology, Inc. Danvers, MA	9205
Anti‐Tuberin	1:2000	Cell Signaling Technology, Inc. Danvers, MA	3612
Anti‐Tuberin		ProteinTech Rabbit Polyclonal	20004‐1‐AP
Biotinylated Dolichos biflorus agglutinin (DBA)	1:300	Vector Laboratories, Burlingame, CA, USA	RL‐1032
Biotinylated Lotus Tetragonolobus Lection (LTL)	1:2,000	Vector Laboratories, Burlingame, CA, USA	B‐1325‐2
Goat polyclonal Anti‐PRR	1:50	Origene Technologies; Rockville, MD	
HMB‐45	1:40	Thermo Scientific, Waltham, MA	MA5‐16712
Mouse monoclonal Anti‐AQP2	1:5,000	Dr. A. Blanchard, Paris‐Descartes University	
Mouse monoclonal Anti‐H+ATPase	1:25	Dr. S. Holliday, University of Florida	
Mouse monoclonal Anti‐PCNA	1:100	Santa Cruz Biotechnologies; Dallas, TX	SC‐56
Phospho‐S6 Ribosomal Protein	1:5,000	Cell Signaling Technology, Inc. Danvers, MA	2211
Rabbit polyclonal Anti‐H^+^ATPase	1:100	Dr. M. Soleimani, University of Cincinnati	
Rabbit polyclonal Anti‐NBCe1	1:40	Dr. M. Soleimani, University of Cincinnati	
Rabbit polyclonal Anti‐NCC	1:60	Stressmarq Biosciences; Victoria, BC, Canada	SPC‐402
Rabbit polyclonal Anti‐NHE3	1:60	Dr. Alicia McDonough, University of California at Los Angeles	
Rabbit polyclonal Anti‐Pendrin	1:15	Generated by Soleimani	
S100	1:100	Abcam, Cambridge, MA	ab52642

### Immunofluorescence labeling

Single and double immunofluorescence labeling was performed using established protocols (Xu et al. 2011; Zahedi et al. 2013; Barone et al. 2018; Varasteh Kia et al. 2018). Briefly, animals were euthanized with an overdose of pentobarbital sodium, and kidneys were perfused with saline followed by 4% paraformaldehyde removed, cut in tissue blocks, and fixed in 4% paraformaldehyde solution overnight at 4°C. The tissue samples were preserved in 70% ethanol, paraffin embedded and 5‐*μ*m sections. For staining, after rehydration and sodium citrate antigen retrieval sections were incubated overnight at 4°C with the primary antibodies (Table 1). Sections were then washed in PBS and incubated with Alexa Fluor conjugated secondary antibodies (Invitrogen, Eugene, OR) for one hour at room temperature. Sections were washed in PBS, dried and cover slips were applied using Vectashield Mounting Medium (Vector Labs, Burlingame, CA).

Immunofluorescence microscopic analysis of tissue sections was performed on a Zeiss Axio Imager.M2 Microscope. Images were acquired using Ziess Axiocam 506C digital camera. Images were processed using Zeiss Zen 2012 software. For each figure, the settings for each panel were identical. For all immunofluorescence studies, more that 95% of the slide had the reported pattern of staining.

### Multispectral imaging of kidney tissue

Kidney from *Ren‐1c‐Cre*,* fl‐Tsc1* or *fl‐Tsc2*, Confetti were excised immediately after euthanasia of the mice. One‐third part of both kidneys were cut into ~1 mm thick sections. Individual sections were squashed to ~20 nm thickness between two glass slides. The tissue was imaged on an inverted Nikon Eclipse Ti fluorescent microscope equipped with a Nuance FX multispectral camera. The four fluorescent proteins encoded by Confetti were identified in the tissue based on reference spectra for each proteins. The remaining kidney parts were fixed in paraformaldehyde, embedded in OCT, and cut in a cryotome into 5‐μm thick sections. The sections were analyzed by multispectral imaging as done for the fresh tissue squashes.

### Development of Tsc‐mutant cell lines

We disrupted either the *Tsc1* or *Tsc2* genes in principal cells using CRISPR/Cas9 genome editing as previously described (Siroky et al. 2017a). A CRISPR plasmid with constitutive green fluorescent protein (GFP) expression and containing guide RNA sequences was constructed by the Cincinnati Children's Hospital Medical Center (CCHMC) Transgenic Animal Genome Editing Core Facility. The guide RNA sequences were selected using algorithms in Benchling.com for on‐target (Doench et al. 2014, 2016) and accounting for off‐target (Hsu et al. 2013) sites. For both the *Tsc1* and *Tsc2* genes, exon 4 was targeted. We used Lipofectamine 3000 (Thermo Fisher Scientific) to transfect mIMCD‐3 cells with these plasmids. At 2 days after transfection, single GFP‐positive cells were sorted into separate wells for expansion (FACSAria II, BD Biosciences, San Jose, CA, located at the CCHMC Research Flow Cytometry Core). After expansion, extracted genomic DNA (GeneJET Genomic DNA Purification Kit, catalog no. K0721, Thermo Fisher Scientific) was used in a polymerase chain reaction (PCR; Phusion Hot Start II DNA polymerase, catalog no. F549, Thermo Fisher Scientific) to amplify the targeted region. The resulting PCR products were screened for loss of the AflII restriction site for Tsc1, and SphI restriction site for Tsc2. PCR products of restriction site mutants were purified (GeneJet PCR Purification Kit, Thermo Fisher Scientific) and sequenced (CCHMC DNA Sequencing and Genotyping Core). For each gene, we selected clones that had, in both alleles, a frame‐shifting mutation that resulted in an early stop codon.

### Clonality assay

A modified clonality assay was used as previously described (Nomura et al. 2004). DNA was extracted from cortical tissues of wild‐type and cyst walls of 11‐week *Aqp2CreTsc2* female‐mice using commercial kit (PureLink, Thermo Fisher Scientific). A total of 50 ng of DNA were digested first with 1.7U of BfaI for 1‐h followed by heat inactivation. This was followed by digestion with either 2U of methyl‐sensitive HpaII or 4U methyl‐insensitive MspII as a control in total of 15 μL reaction. Similar protocol followed for undigested sample in which no HpaII or MspI was added. A total of 2 μL of undigested, HpaII and MspI digestion product were used to amplify the seventh CpG island of mouse X‐linked *pgk‐1* promoter with following primers: CGCTGTTCTCCTCTTCCTCA (forward) and GGACGCAGAAAA‐GCAAACTC (reverse). PCR products were separated in 1% agarose gel.

### Extracellular vesicle isolation

Cells were serum starved for 24 hours. Serum‐free media were replaced and harvested after 24‐hour incubation. The conditioned media were centrifuged at 2000 g, 4°C for 30 min to remove cells and debris. The supernatants were transferred to new tubes with a half volume of total Exosome Isolation reagent (Cat. # 4478359, Thermo fisher Scientific). The tubes were mixed well and incubated overnight at 4°C, and centrifuged at 10,000 g for 1 h at 4°C. The supernatant was aspirated and discarded, and the pelleted extracellular vesicles were resuspended in phosphate‐buffered saline.

### Human imaging studies

Following IRB approval, sequential de‐identified patient imaging was evaluated for cystic burden by cyst count.

### Statistics

All statistical analyses were performed using the R package version 3.1.0 (Team RC 2018). Cox proportional hazard models were performed using the Survival package (version 2.38‐3; (Therneau 2018; Therneau and Grambsch 2000)). Animals that died due to sacrifice or disease‐related death were censored from the analyses.

## Results

### Mouse models of Tsc cystic kidney disease

**Figure 1:**
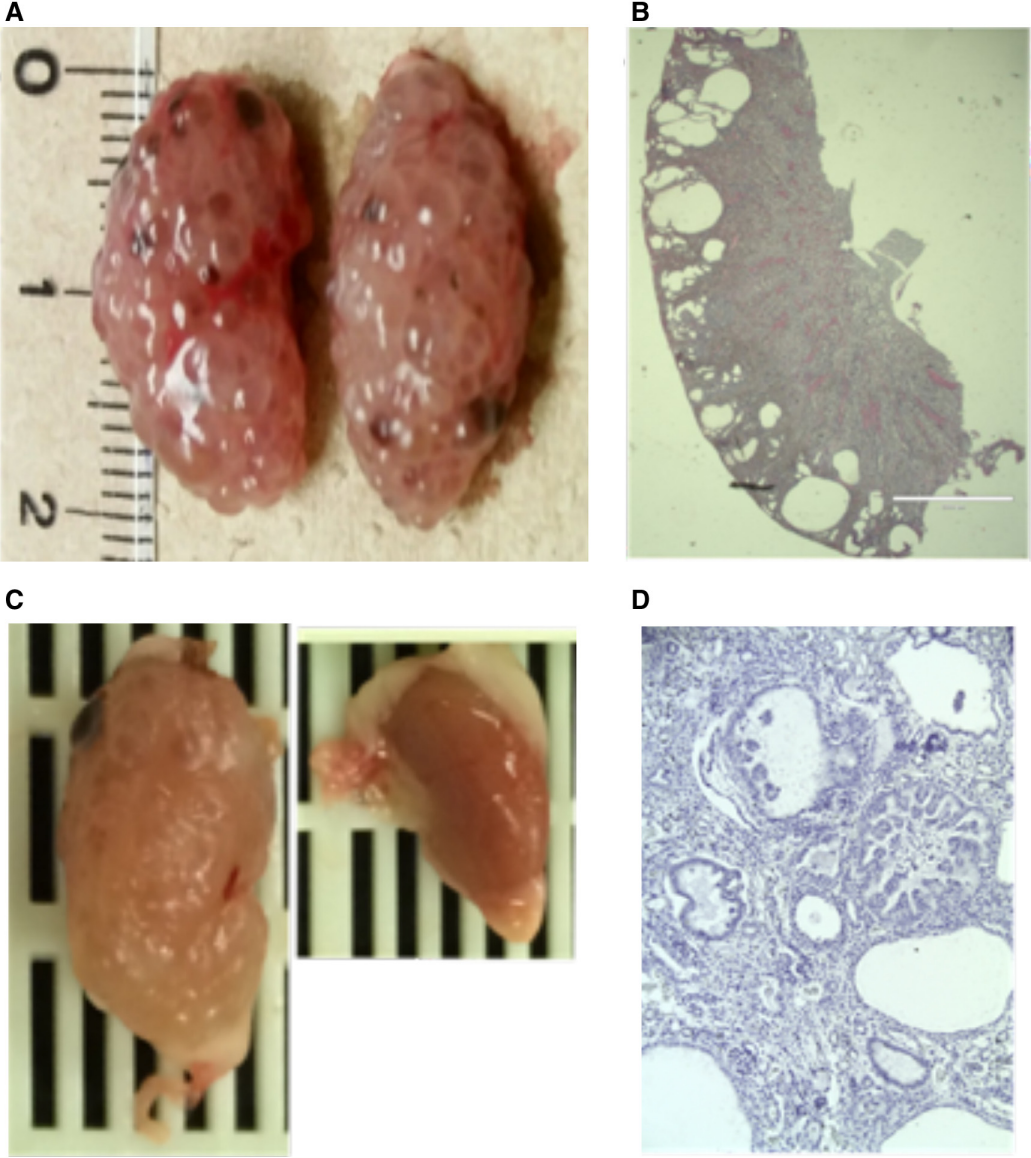
*Tsc* renal cystic disease. (A) *AqpCreTsc2* kidneys at 11 weeks with significant renal cystic disease. (B) Coronal sections of the kidney in figure A. (C) Mouse kidneys from *RenCreTsc1* mouse with unilateral cystic disease. These are on the same size scale as in A. (D) Coronal section of kidney in figure C.

The most severe forms of human TSC cystic disease include polycystic variety associated with the contiguous gene syndrome (Brook‐Carter et al. 1994; Jones et al. 1997) and microcystic disease (Bissler 2018). There is a well‐established association between primary cilia defects and the development of renal cystic disease. To create a mouse model that would phenocopy these severe TSC renal diseases, we targeted the primary cilia expressing renal collecting duct principal cell. To achieve this, we disrupted the floxed *Tsc2* gene in the kidney using by aquaporin‐2 (*Aqp2*) promoter to drive Cre‐recombinase (*Aqp2Cre*) expression. By crossing these *Aqp2Cre* transgenic mice with floxed *Tsc2* mice, we generated the *Aqp2CreTsc2* mouse. These mice have relatively normal kidneys at birth but develop florid cystic disease by week 11 (Fig. 1A). At 11 weeks, mice that do not harbor the *Aqp2Cre* have kidneys that are 1 cm long, while mice that do express the recombinase (*Aqp2CreTsc2*) have kidneys that are twice this length and have visible cysts on the surface (Fig. 1A).

Coronal sections of the diseased kidney (Fig. 1B) revealed a cortical cyst pattern. The cortical cysts were not matched by medullary cysts over time, despite the fact that the principal cell density increases along the collecting duct into medulla. The *Aqp2CreTsc2 mice* had a reduced median survival of 147 days and did not develop angiomyolipomata.

We posited that there is a causal relationship between angiomyolipomata and TSC renal cystic disease, so our second model focused on cystic disease caused by disrupting the *Tsc* axis in a putative cell of origin of angiomyolipomata. Recent evidence suggests that angiomyolipomata arise from a subpopulation of renal vascular pericytes (Siroky et al. 2014); therefore, we used the *Ren1cCre* to disrupt the floxed *Tsc1* allele (*Ren1cCreTsc1*). Although *Ren1cCre* recombinase is expressed in the renal pericyte compartment, the animals most often develop cystic disease. The disease appeared to be more commonly unilateral, without a side preference. These mice also had a 25% reduction in their litter sizes (*P* = 0.02 via Fisher's Exact Test), suggesting that bilateral disease compromised survival. The affected kidney in these animals was larger than the grossly unaffected contralateral kidney (Fig. 1C). Histologically the affected kidney exhibited significant renal cyst epithelial cell enlargement, some cystic epithelia were hyperplastic, and occasional prominent nucleoli were identified (Fig. 1D). These mice grew at a normal rate, with normal body weight and no apparent gross developmental defects, but showed lethality at a median of 85 days, essentially during adolescence.

### Tsc genes in cystic epithelium remain intact

**Figure 2:**
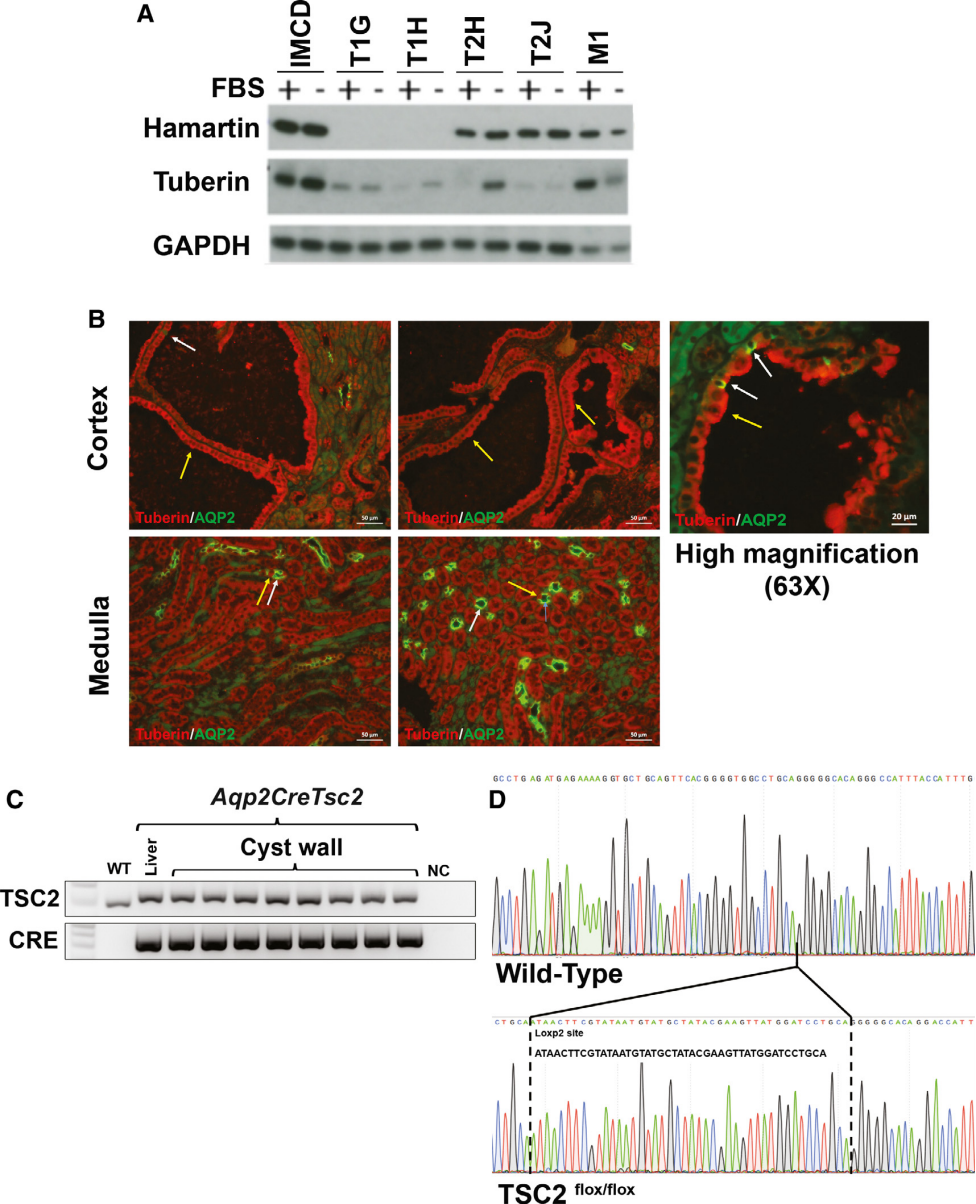
Cystic epithelium express tuberin and have not undergone Cre mediated recombination. (A) Western blot of hamartin and tuberin expression in IMCD cells and derived hamartin‐ (T1G and T1H) and tuberin‐knockdown cells (T2H ad T2J). Knock‐down of hamartin is known to reduce tuberin expression (Barone et al. 2018). (B) Section of mutant kidney cortex and medulla. While the principal cells should express aquaporin‐2 and not tuberin, other cells that do not express aquaporin‐2 should maintain tuberin expression. Cystic epithelium was restricted to the cortex and continued to express tuberin (yellow arrows). At higher magnifications (63X) some aquaporin expressing cells were identified (white arrows). The medullary cells, believed to be medullary collecting ducts, robustly expressed aquaporin‐2. (C) Cystic epithelium exhibits a PCR band that is the correct size for the non‐recombined *lox*P2 site. (D) Sequencing these bands reveal that they contain the *lox*P2 site indicating that they did not undergo Cre mediated recombination.

Because previous murine models of *Tsc* renal cystic disease do not show convincing “second hit” mechanism for the *Tsc* renal disease (Onda et al. 1999; Wilson et al. 2006), and human TSC‐associated cysts express hamartin and tuberin (Bonsib et al. 2016), we wished to ascertain the *Tsc* gene integrity in cystic epithelium in our models. To assess if the cysts exhibited the loss of heterozygosity as described in the angiomyolipomata, we used immunohistochemistry as well as a genetic analysis for *Tsc2* recombination in the cysts from the *Aqp2CreTsc2* mice. To validate the results, we used two different commercially available antibodies with publicly available validation for tuberin (ProteinTech 2018; Cell_Signaling_Technology 2018) western blot analysis and a polymerase chain reaction assay to probe for recombination by the maintenance of a *Lox*P2 site. We assayed one of the antibodies by western blot to assure that we got similar results to reported data (Cell Signaling, see Table 1). To generate target cell lines, we used a parental cell line from mouse inner medullary collecting ducts (IMCD), and isogenic cell lines that were modified to disrupt either *Tsc1*(T1H and T1J) or *Tsc2* (T2H and T2J) using CRISPR/CAS9 technology (Fig. 2A). The effect of harmartin expression on tuberin expression has previously been reported (Benvenuto et al. 2000). We identified tuberin expression in cystic epithelium with both antibodies. Furthermore, we found that the expression of tuberin was not seen in the less common cell that expressed aquaporin‐2 (Fig. 2B). The cortical expression and cysts was distinct from the expression in the medulla, where again tuberin or aquaporin‐2 expression were mutually exclusive, but aquaporin‐2 expressing cells were much more numerous. To genetically assess *Tsc2* gene recombination, we used a PCR approach to see if the *lox*P2 site was retained. The cystic epithelium demonstrated the same size band comparing DNA from liver and cyst wall epithelium (Fig. 2C). Sequencing these bands demonstrated that they retain the sequence and had not undergone Cre‐mediated recombination (Fig. 2D).

**Figure 3:**
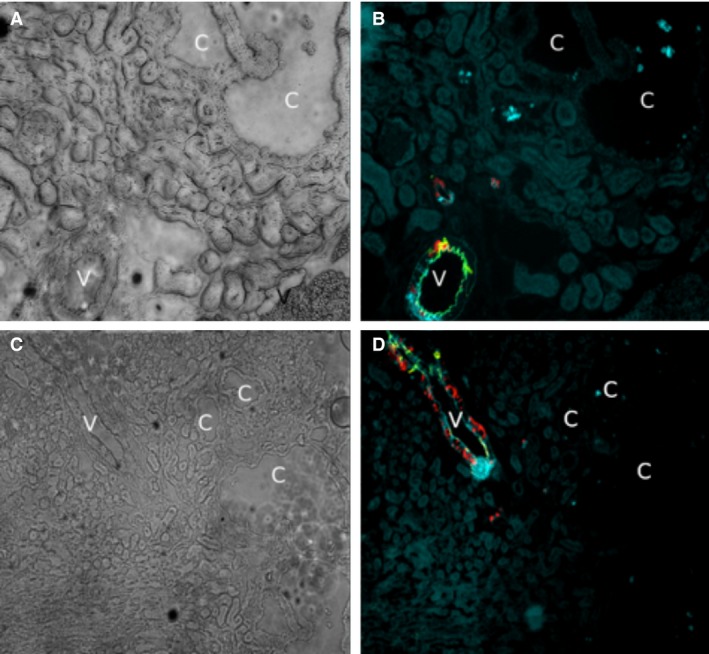
The Tsc1 gene has not undergone recombination in the cystic epithelium of the *Ren1cCreTsc1* model. (A) Brightfield of *Ren‐1c‐CreTsc1* kidney arteriole, denoted by “v”, and cysts, denoted by “c”. (B) Fluorescence of the same tissue in panel A demonstrating vascular pericyte derived fluorescence, while cysts are devoid of signal. (C) Brightfield of arteriole and cysts. (D) Fluorescence of tissue in C with only vessel fluorescing.

These results lead us to probe for recombination in the cysts of the *Ren1cCreTsc1* mice. The cystic disease in the *Ren1cCreTsc1* model has more numerous and smaller cysts (Fig. 1C and D), so directly obtaining cystic tissue would be more prone to contamination by DNA from non‐cystic cells. Instead, we probed for *Ren1cCreTsc1* cystic *Tsc1* deletion by breeding the *Ren1cCreTsc1* onto the “confetti” reporter background (Fig. 3A–D). We utilized this technique to evaluate genetic status of the cysts in *Ren1cCreTsc1* affected kidneys. While we identified clear fluorescence in arterioles, indicating Cre‐mediated recombination, we could not identify fluorescence in the cystic epithelium in any of the slides screened. Thus, like in the *Aqp2CreTsc2* model, the genetic evidence corroborates the immunohistochemical evidence that *Tsc* genes were intact in the cysts in these models.

### Cellular composition of Tsc cystic epithelium

**Figure 4:**
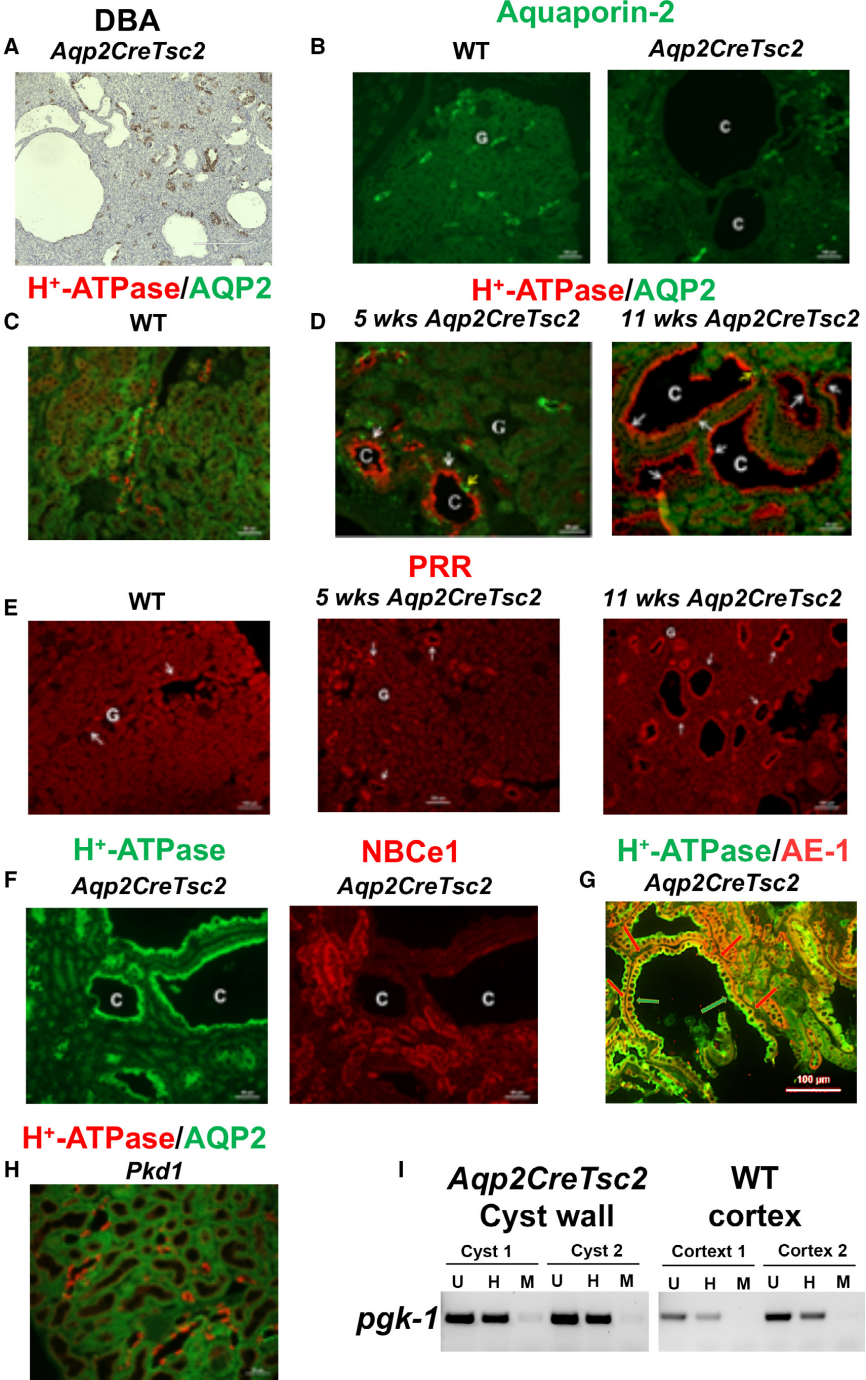
Cyst epithelial phenotypes in *AqpCreTsc2* mice. (A) DBA staining in cyst epithelia in *AqpCreTsc2 (*Tsc‐2 KO) mice. (B) Expression of AQP‐2 in kidneys of WT (left) and *AqpCreTsc2 (*Tsc‐2 KO) mice (right). (C) Double immunofluorescence labeling with AQP‐2 (green) and H^+^‐ATPase (red) antibodies in normal kidney (merged image). (D) Double immunofluorescent labeling with AQP‐2 (green) and H^+^‐ATPase (red) in kidneys of 5 weeks (left panel) and 11 weeks old (right panel) *AqpCreTsc2* mice (merged image). (E) Expression of prorenin receptor (PRR) in kidneys of *AqpCreTsc2* mouse. Left panel: normal kidney; Middle panel: 5 weeks old *AqpCreTsc2* mice. Right panel: 11 weeks old *AqpCreTsc2* mice. (F) Double immunofluorescent labeling with NBC‐e1 (right panel) and H^+^‐ATPase B subunit (left panel) in kidneys of 11 weeks old *AqpCreTsc2* mice. (G) Cyst double labeling with H^+^‐ATPase (green arrow) and AE‐1 (red arrow), additional evidence that cystic epithelium consists of type A intercalated cells. (H) Double immunofluorescence labeling with H^+^‐ATPase and AQP‐2 in kidneys of *Pkd1* mouse (merged image). (I) PCR products of female mouse *pgk*‐1 promoter region on the X chromosome, for undigested sample, U, methyl‐dependent *Hpa* II digested, H, and methyl‐independent *Msp*I digested samples from WT and *AqpCreTsc2* mice. Cystic cell DNA is not clonal as band intensity is diminished by > 25% when input DNA is pre‐digested with *Hpa* II. C: cyst, G: Glomerulus

To characterize the renal cystic epithelial and gain insight into the cystogenic pathways and/or mechanisms at play in these models, we used both lectin and immunofluorescent staining. Multiple cystic epithelia histochemically stained diffusely with *Dolichos biflorus* agglutinin (DBA), which predominantly labels the collecting duct, while a few cysts were not stained (Fig. 4A). To identify the nephron segment(s) and the cell types comprising the cyst epithelium, single and double immunofluorescence labeling experiments were performed using antibodies against transporters, channels or molecules with specific nephron segment distribution. We first probed for principal cells labeling in renal collecting ducts of wild‐type (left panel) and *Aqp2CreTsc2* mice (right panel) using anti‐aquaporin‐2 (AQP‐2) immunofluorescent staining (Fig. 4B). AQP‐2 expression was present in non‐cystic tubules but surprisingly was absent in cyst epithelial cells in *Aqp2CreTsc2* mice (Fig. 4B, right panel). The labeling in wild‐type kidney is consistent with AQP‐2 expression in principal cells (Fig. 4B, left panel). A double immunofluorescence labeling with AQP‐2 (green) and H^+^‐ATPase (red), a marker of intercalated cells, in a normal mouse kidney shows multiple principal cells (AQP‐2 positive) interspersed with intercalated cells (H^+^‐ATPase positive) in wild‐type cortical collecting ducts (Fig. 4C). Using AQP‐2 and H^+^‐ATPase double immunofluorescence staining in the *Aqp2CreTsc2* kidney revealed an age‐dependent change. Figure 4D demonstrates double immunofluorescent labeling with AQP‐2 (green) and H^+^‐ATPase B subunit (red) in kidneys of 5 weeks (left panel) and 11 weeks old (right panel) *Aqp2CreTsc2* mice. As shown, 5 weeks old *Aqp2CreTsc2* mice exhibit early signs of cyst development in their kidneys (depicted with the letter “C”). Several principal cells and numerous intercalated cells are present in cyst epithelium as verified by the expression of AQP‐2 (green) and H^+^‐ATPase (red), respectively, in distinct cells. Interestingly, the H^+^‐ATPase‐positive cells (white arrows) are more abundant compared to AQP‐2 positive cells (yellow arrows) in cyst epithelium. Kidneys of 11 weeks old *Aqp2CreTsc2* mice (Fig. 4D, right panel) show more frequent and larger cysts in the cortex and display robust and almost uniform expression of H^+^‐ATPase along the apical membrane of cyst epithelium. There are very few AQP‐2 positive cells in cyst epithelia in 11 weeks old *Aqp2CreTsc2* mice (Fig. 4D, right panel). These changes are consistent with the proliferation of A‐intercalated cells and gradual disappearance of principal cells in cyst epithelium.

We were intrigued by the H^+^‐ATPase in the intercalated cells because in the kidney, the prorenin receptor (PRR) co‐localizes with H^+^‐ATPase in intercalated cells and functions as a receptor for renin. Pro‐renin, a critical chaperone for the assembly of H^+^‐ATPase subunits, regulates cell proliferation via the mitogen activated protein kinases ERK1/2 cascade and is a crucial component of Wnt pathways (Kaneshiro et al. 2006; Cao and Feng 2013; Seva Pessoa et al. 2013; Sihn et al. 2013; Danser 2015; Ramkumar and Kohan 2016a,b; Peters 2017). There is robust and uniform PRR expression on the apical membrane of cyst epithelium in 11 weeks old *Aqp2CreTsc2* mice (Fig. 4E, right panel) and parallels the H^+^‐ATPase expression pattern, as does the PRR expression in the 5‐week old *Aqp2CreTsc2* mice (Fig. 4E, middle panel). PRR expression in control animals is shown for comparison (Fig. 4E, left panel).

To determine whether other nephron segments or tubular cells contribute to cyst epithelium, we performed double labeling with the Na^+^:HCO_3_^−^ cotransporter NBC‐e1 (red fluorescence), which primarily labels the basolateral membrane of proximal tubule and thick ascending limb of Henle (TALH), and H^+^‐ATPase (green fluorescence), which is strongly expressed in intercalated cells. NBC‐e1 (Fig. 4F, right panel) was detected in normal, non‐cystic nephron segments, but was completely absent in cyst epithelial cells in adult *Aqp2CreTsc2* mice. The H^+^‐ATPase showed a very strong and uniform labeling in cyst epithelial cells (Fig. 4F, left panel). The Na‐Cl cotransporter NCC, a marker of distal convoluted tubule, and pendrin, a marker of B‐intercalated cells, did not show any labeling in cyst epithelia (Data no shown). Additional staining for the sodium‐hydrogen exchanger‐3, a marker of proximal tubule and thick ascending limb of Henle, failed to identify this transporter in the cystic epithelium (Data not shown). The Na^+^‐K^+^ ATPase showed normal expression in non‐cystic nephrons but displayed minimal basolateral labeling in cyst epithelial cells (Data not shown). Cystic epithelium does express type A intercalated cell localization of H^+^‐ATPase on the apical surface and AE‐1 on the basolateral surface (Fig. 4G).

Because we identified a new cystogenic mechanism, we wished to compare Tsc renal cystic disease to *Pkd1* renal disease. Kidney sections from a *Pkd1*‐mutant mouse (generated using a PAX8 cre mouse and a generous gift from Dr. Stephen Somlo) were examined for the expression of H^+^‐ATPase and AQP‐2. Double immunofluorescence studies demonstrate that in *Pkd1*‐mutant mice, cysts originating from the cortical collecting duct contain both principal cells and intercalated cells (Fig. 4H).

We used female mice and X‐chromosome inactivation to assess if type A intercalated cells in cystic epithelia results from expansion of single cell (i.e., monoclonal) or multiple cells (polyclonal). Figure 4I shows a greater than 25% reduction in methylated‐*pgk1* locus on the X chromosome in cortical tissues of wild type and cystic epithelia of *Aqp2CreTsc2 female* mice, more strongly supporting a polyclonal nature of the cystic epithelium.

**Figure 5:**
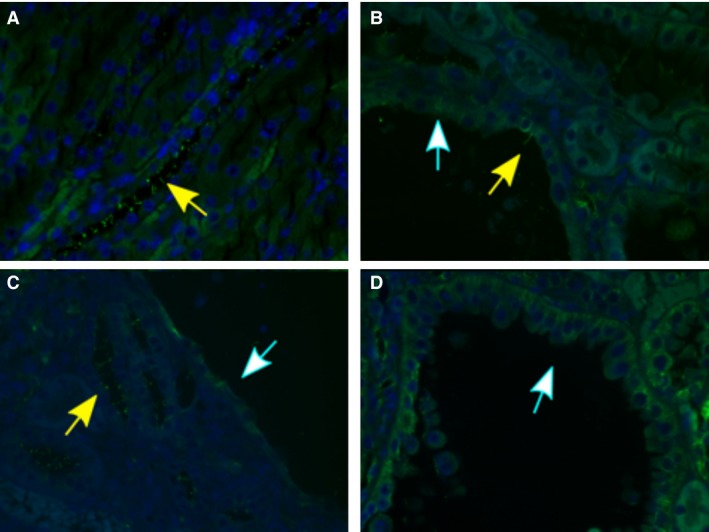
*AqpCreTsc2* cystic epithelium exhibit much fewer primary cilia than adjacent tubules. (A) Immunofluorescence of acetylated tubulin (yellow arrow) easily identify primary cilia in a medullary collecting duct. (B‐D) Cystic epithelium exhibit a significant suppression of primary cilia (white arrow) but does exhibit occasional cilia (yellow arrow).

The absence of labeling with AQP‐2, pendrin, NBC‐e1, NCC or NHE‐3 antibodies in renal cystic epithelium of *Tsc2*‐specific knockout mice indicated that the cysts were not comprised of the targeted principal cells, nor the B‐intercalated cells, proximal tubule cells, thick ascending limb of Henle cells or distal convoluted tubule cells. The H^+^‐ATPase apical location correlated very well with the cyst fluid pH that ranged from 5 to 5.6, and was most compatible with the cystic epithelium exhibiting an A‐intercalated cell phenotype. This cell of origin is also important as the intercalated cells do not express a primary cilia, and the cysts in out model also fail to express significant cilia (Fig. 5).

**Figure 6:**
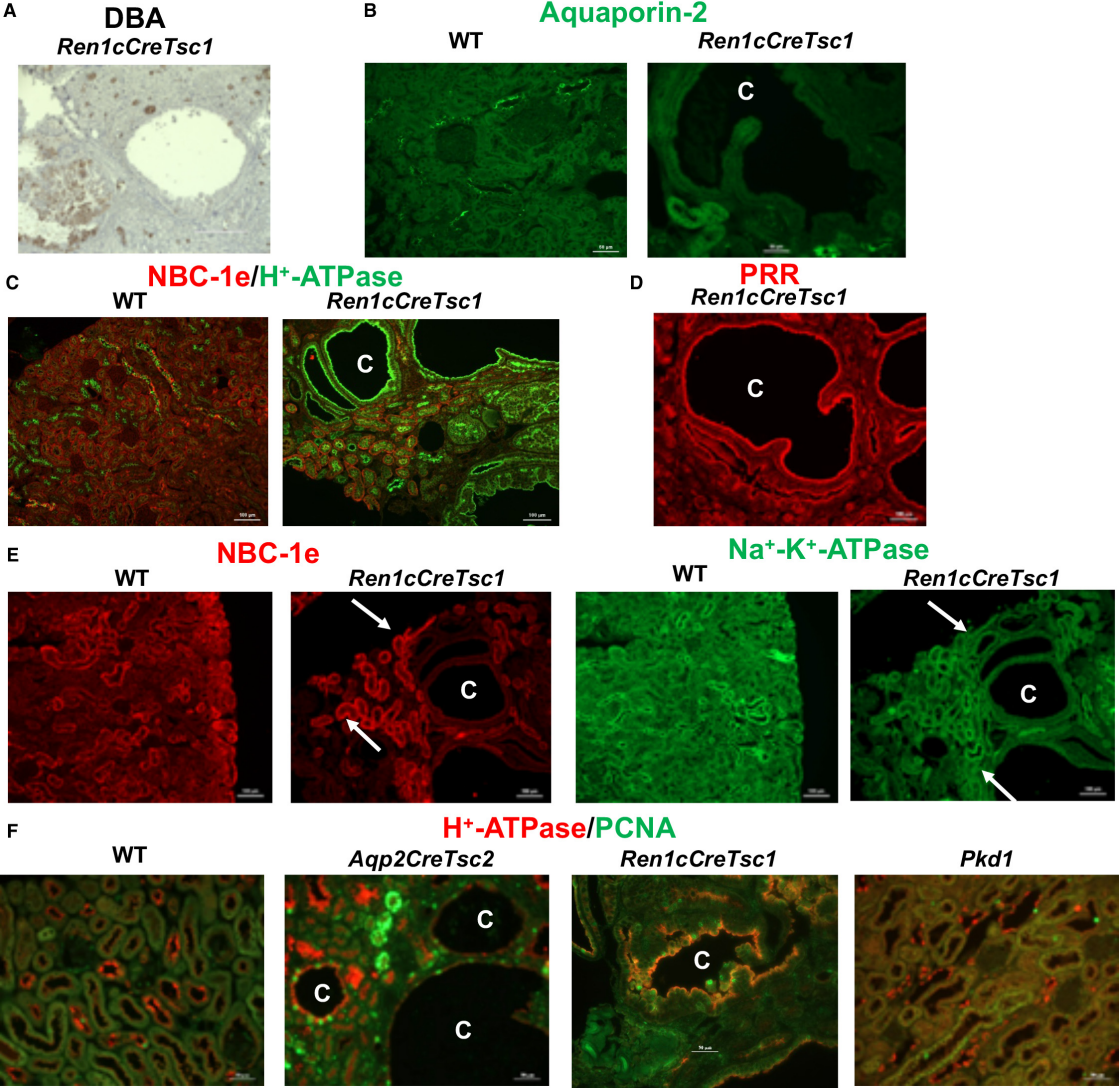
Cyst epithelial phenotypes in *Ren1cCreTsc1* mice. (A) DBA staining in cyst epithelia in *Ren‐1c‐ CreTsc1* (Tsc‐1 KO) mice. (B) Expression of AQP‐2 in kidneys of WT and *Ren1cCreTsc1* mice. (C) Double immunofluorescence labeling (merged image) with H^+^‐ATPase (green) and NBC‐e1 (red) antibodies in normal kidney (left) and adult *Ren1cCreTsc1* mice (right). (D) Expression of PRR in kidneys of *Ren1cCreTsc1* mice. (E) Double immunofluorescence labeling with Na^+^‐K^+^ ATPase (green) and NBC‐e1 (red) in normal kidney (first and third panels) and *Ren1cCreTsc* mice (second and fourth panels). (F) Double immunofluorescence labeling with H^+^‐ATPase (red) and PCNA (Proliferating Cell Nuclear Antigen) antibodies (merged image) in kidneys WT, AqpCreTsc2, *RenCreTsc1,* and *Pkd1* mice.

Because of the novel cystogenic process in the *Aqp2CreTsc2* model, we conducted similar studies in the *Ren1cCreTsc1* model. As with the *Aqp2CreTsc2* mouse model, the DBA staining was detected in most but not all cysts in the *Ren1cCreTsc1* model (Fig. 6A). Similar to *Aqp2CreTsc2* mice, the *Ren1cCreTsc1* mice did not show any expression of AQP‐2 on the apical membrane of cyst epithelia (Fig. 6B, right panel). The expression of AQP‐2 in cortical collecting duct in a normal kidney is shown for comparison (Fig. 6B, left panel). The double labeling with NBC‐e1 and H^+^‐ATPase revealed a strong and uniform expression of H^+^‐ATPase on the apical membrane of cyst epithelia but did not demonstrate any labeling with NBC‐e1, a marker of proximal tubule and thick ascending limb of Henle cells (Fig. 6C, right panel). The expression of NBC‐e1 in normal kidney **(**Fig. 6C, left panel**)** and in non‐cystic kidney tubules in *RenCreTsc1* is shown (Fig. 6C, right panel). The cystic epithelia showed a strong and uniform expression of PRR on their apical membrane (Fig. 6D). The double labeling with Na^+^‐K^+^ ATPase and NBC‐e1 indicates basolateral expression of Na^+^‐K^+^‐ATPase in multiple nephron segments in normal kidney (Fig. 6E, first and third panels). In *Ren1cCreTsc1* mice, the Na^+^‐K^+^‐ATPase exhibits strong basolateral expression in non‐cystic tubules and mild expression in cystic epithelia (Fig. 6E, fourth panel). The basolateral Na^+^:HCO_3_^−^ cotransporter NBC‐e1 shows no expression in cystic epithelia but shows normal expression in non‐cystic epithelia (Fig. 6E, second panel). There was no labeling with NCC, pendrin, or NHE‐3 antibodies in renal cysts, indicating the lack of B‐intercalated cells, principal cells, distal convoluted tubule cells, proximal tubule cells or thick ascending limb of Henle cells in cyst epithelium adult *Ren1cCreTsc1* mice.

To better establish the role of intercalated cells in cyst epithelia proliferation in these *Tsc* knockout mice, we performed double labeling with H^+^‐ATPase and proliferating cell nuclear antigen (PCNA) in kidneys of *Aqp2CreTsc2* and *Ren1cCreTsc1* mice (Fig. 6F). Similar staining was performed on kidneys from *Pkd1* mice (Fig. 6F, fourth panel). For comparison, PCNA labeling in wild‐type mice is shown (Fig. 6F, first panel).

As observed in multiple high magnification images, wild‐type mice showed very few PCNA‐positive cells per each field (Fig. 6F, first panel). However, the H^+^‐ATPase‐expressing epithelial cells lining the cysts in *Aqp2CreTsc2* mice showed numerous PCNA‐positive cells (Fig. 6F, second panel**)**. Similarly, the cyst epithelium in *Ren1cCreTsc1* mice showed many H^+^‐ATPase expressing cells that also displayed positive staining for PCNA (Fig. 6F, third panel), a pattern very similar to *Aqp2CreTsc2* mice. The cells lining the cysts in *Pkd1* mice showed a relative increase in the number of PCNA‐positive cells in cyst epithelium as compared to wild‐type mice (Fig. 6F, fourth panel). However, the number of PCNA‐positive cells in *Pkd1* mice was significantly less than that in *Aqp2CreTsc2* or *Ren1cCreTsc1* mice. Furthermore, only a minority of PCNA‐expressing cells showed co‐labeling with H^+^‐ATPase (Fig. 6F, fourth panel).

Our results consistently indicate that A‐intercalated cells with an unrecombined *Tsc* gene are induced or reprogrammed to form cysts in both *Aqp2CreTsc2* or *Ren1cCreTsc1* models. Basically, the loss of heterozygosity in the small number of cells would be combined with a gain of function mechanism that affects the surrounding genetically unrecombined cells and tissue. The significant difference in A‐intercalated cell expansion in *Tsc* knockout mice versus *Pkd1* mice (results above) highlights the difference in the role of A‐intercalated cells in these two kidney cystic models.

### mTORC1 inhibition response of genetically normal cystic cells

The examination of these two very different murine models of real cystic disease suggest the presence of a common mechanism involving the adoption of a *Tsc*‐mutant phenotype in genetically normal cells that is induced by the loss of *Tsc* function in a small number of adjacent cells. These observations have important implications as far as mechanism of disease and possible response to mTORC1 inhibitors are concerned. Such inhibitors are effective and are approved for the treatment of TSC‐associated renal angiomyolipomata (Bissler et al. 2008, 2013), subependymal giant cell astrocytomas (Franz 2013), and lymphangioleiomyomatosis (Bissler et al. 2008; McCormack et al. 2011) and possibly small cysts in humans (Siroky et al. 2017b). However, unlike the angiomyolipomata in human, these murine models of cystic renal disease involve non‐mutant epithelium (Franz et al. 2018).

**Figure 7:**
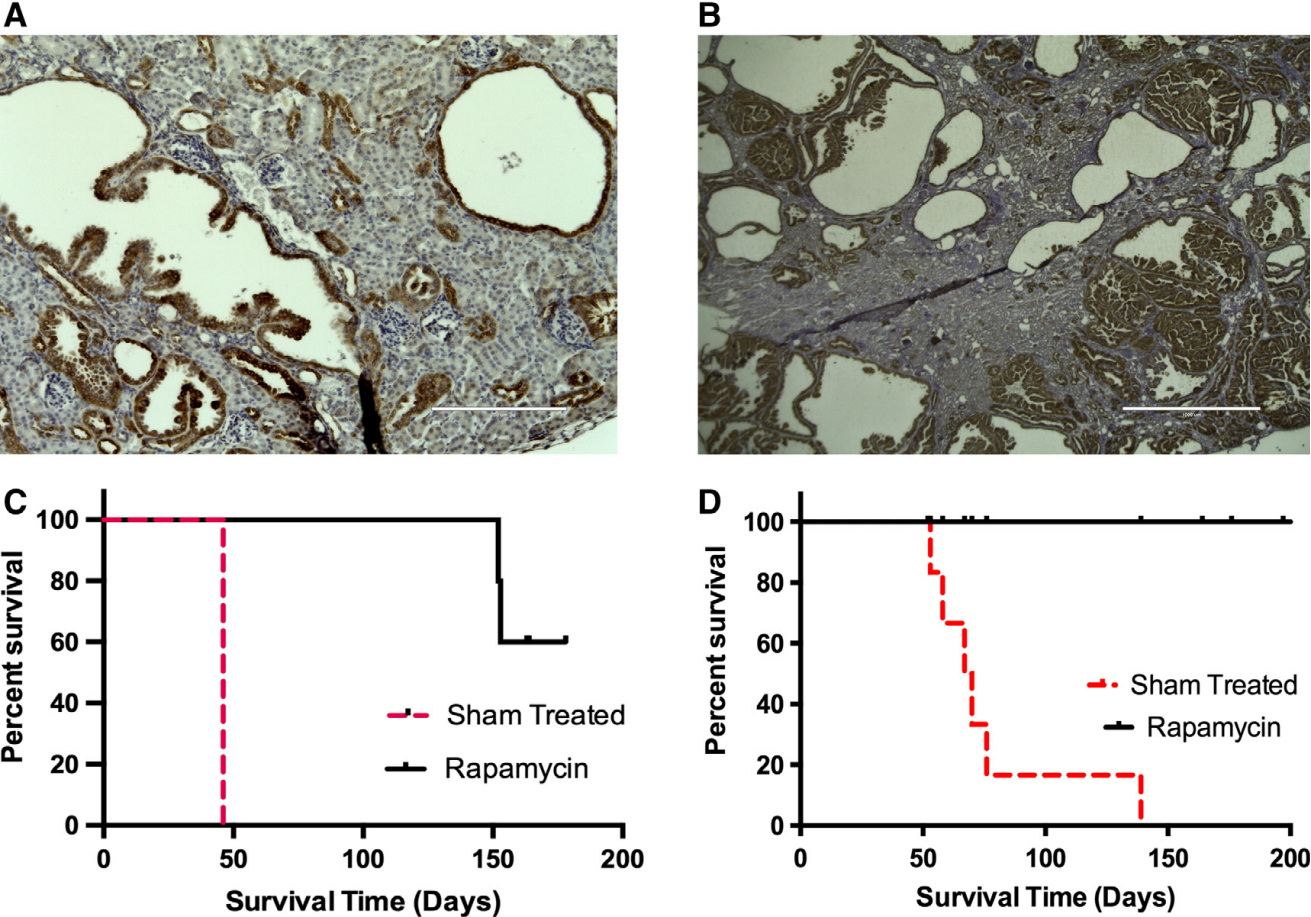
mTORC1 involved in cystic disease. (A) *AqpCreTsc2* cysts stain robustly for phospho‐S6 (bar is 50 *μ*m). (B) *RenCreTsc1* cysts also stain robustly for phospho‐S6 (bar is 1000 *μ*m). (C) mTORC1 inhibition significantly prolongs *AqpCreTsc2* survival compared to sham treated (*P* = 0.0293) (*n* = 5 mice each group). (D) mTORC1 inhibition also significantly prolongs *RenCreTsc1* survival compared to sham‐treated animals (*P* < 0.0001) (*n* = 10 mice each group).

To determine if mTORC1 activation was involved in this new cystogenic mechanism, we first examined the phospho‐S6 staining in the *Aqp2CreTsc2* cystic tissue. There was a uniform increase in phospho‐S6 staining of the cystic epithelium (Fig. 7A). There was also a significant increase staining for phospho‐S6 in the *Ren1cCreTsc1* cystic epithelium (Fig. 7B). These results support the involvement of the mTORC1 pathway in this novel mechanism of cystogenesis, even though the *Tsc* genes are intact. To test whether inhibiting the mTORC1 axis could be therapeutic, we treated animals from both models with rapamycin and used survival as a readout of effect. Administration of rapamycin for the *Aqp2CreTsc2* and the *Ren1cCreTsc1* significantly prolonged the animal survival (Fig. 7C and D).

### Implications for human disease

**Figure 8:**
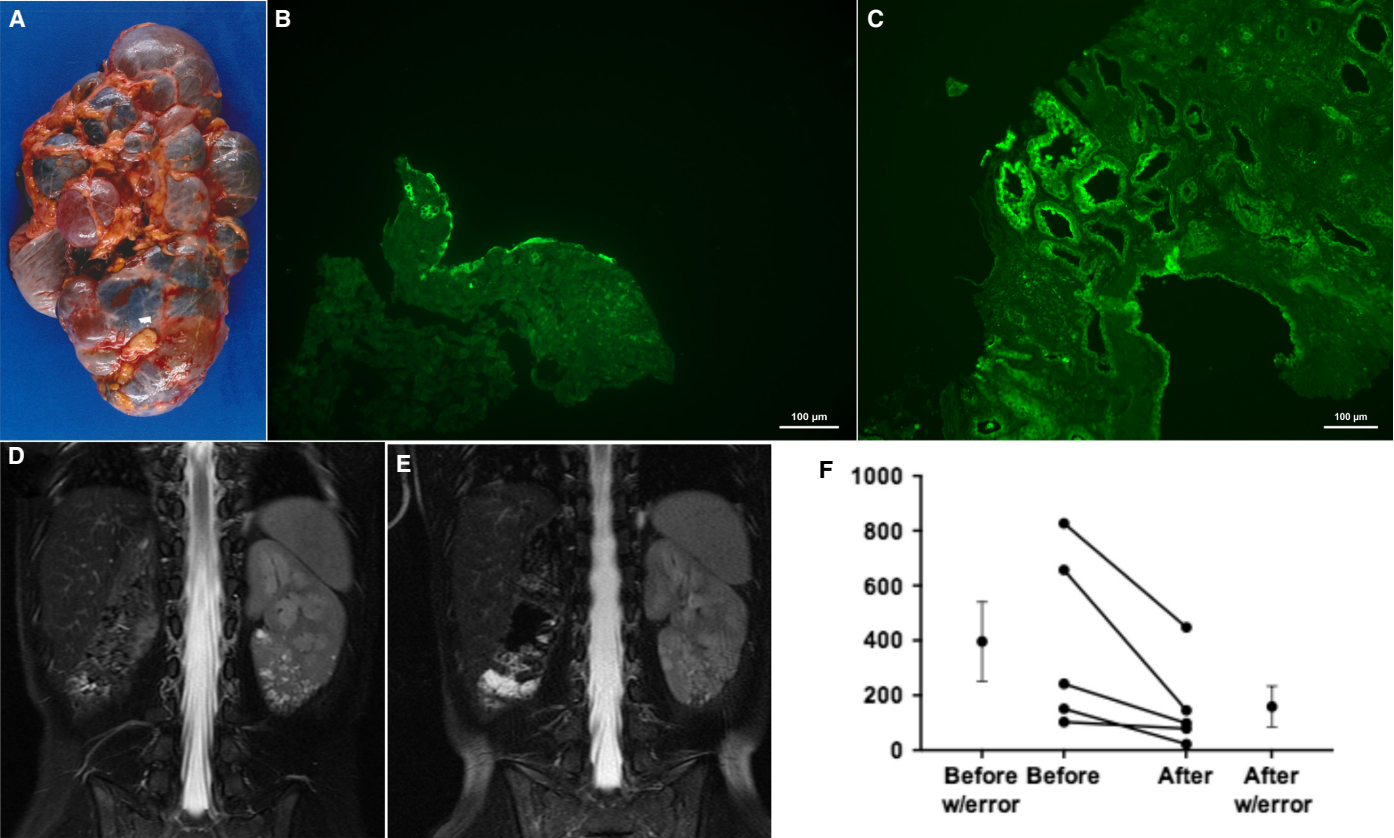
Human Polycystic variety of TSC renal Disease. (A) Human polycystic kidney variety of TSC renal disease at nephrectomy. (B) Cyst epithelium from kidney in panel A exhibiting apical staining with H^+^‐ATPase antibody (bar is 100 *μ*m). (C) Biopsy specimen from patient with TSC‐related microcystic disease exhibiting apical staining with H^+^‐ATPase antibody (bar is 100 *μ*m). (D) MRI imaging (T2 fast spin echo with fat suppression) from patients with TSC cystic disease (white lesions in solitary left kidney) before mTORC1 inhibitor therapy, (E) Patient in panel “D” after one year on drug. (F) Analysis of total kidney cyst count from five patients with cortical cystic disease and focal cystic disease before and after mTORC1 therapy.

The polycystic and the microcystic kidney variety of TSC lead to loss of renal function by adolescence or young adulthood. These forms of TSC renal cystic disease are in the most need of therapeutic intervention. Given the unexpected results reported here, we wished to determine if human disease exhibited a similar pathology and response to mTORC1 inhibition. To test the staining characteristics of human TSC renal cystic disease associated tissue, we used a nephrectomy specimen for the polycystic kidney disease phenotype (Fig. 8A). Similar to the mouse models, the human TSC cystic apical epithelium revealed H^+^‐ATPase staining (Fig. 8B) and demonstrated findings again consistent with cystogenic participation of A‐intercalated cells. A similar finding was also revealed in the staining of the microcystic disease biopsy (Fig. 8C).

While the A‐intercalated cells appear to be involved in human TSC renal cystic disease, therapeutic response to mTORC1 inhibition is not clear. Although one publication suggested a possible response of mild TSC cortical cystic disease to mTORC1 inhibitor therapy (Siroky et al. 2017b), it is not clear that mTORC1 inhibition is useful for severe forms of TSC renal cystic disease. We examined patients with the focal and cortical cystic variants of TSC (Bissler and Kingswood 2018) who were on an mTORC1 inhibitor clinically and found that they also respond to mTORC1 inhibitors by reducing their cystic burden (Fig. 8 D and E). Five TSC patients were identified that were placed on an mTORC1 inhibitor with either focal or cortical cystic kidney disease for at least 1 year. Total renal cystic count of the cysts were performed prior to therapy and after at least 1 year, and a significant reduction (*P* < 0.025 ratio paired *t*‐test) of cystic burden was identified (Fig. 8F).

### Possible role of extracellular vesicles in disease phenotype induction

**Figure 9:**
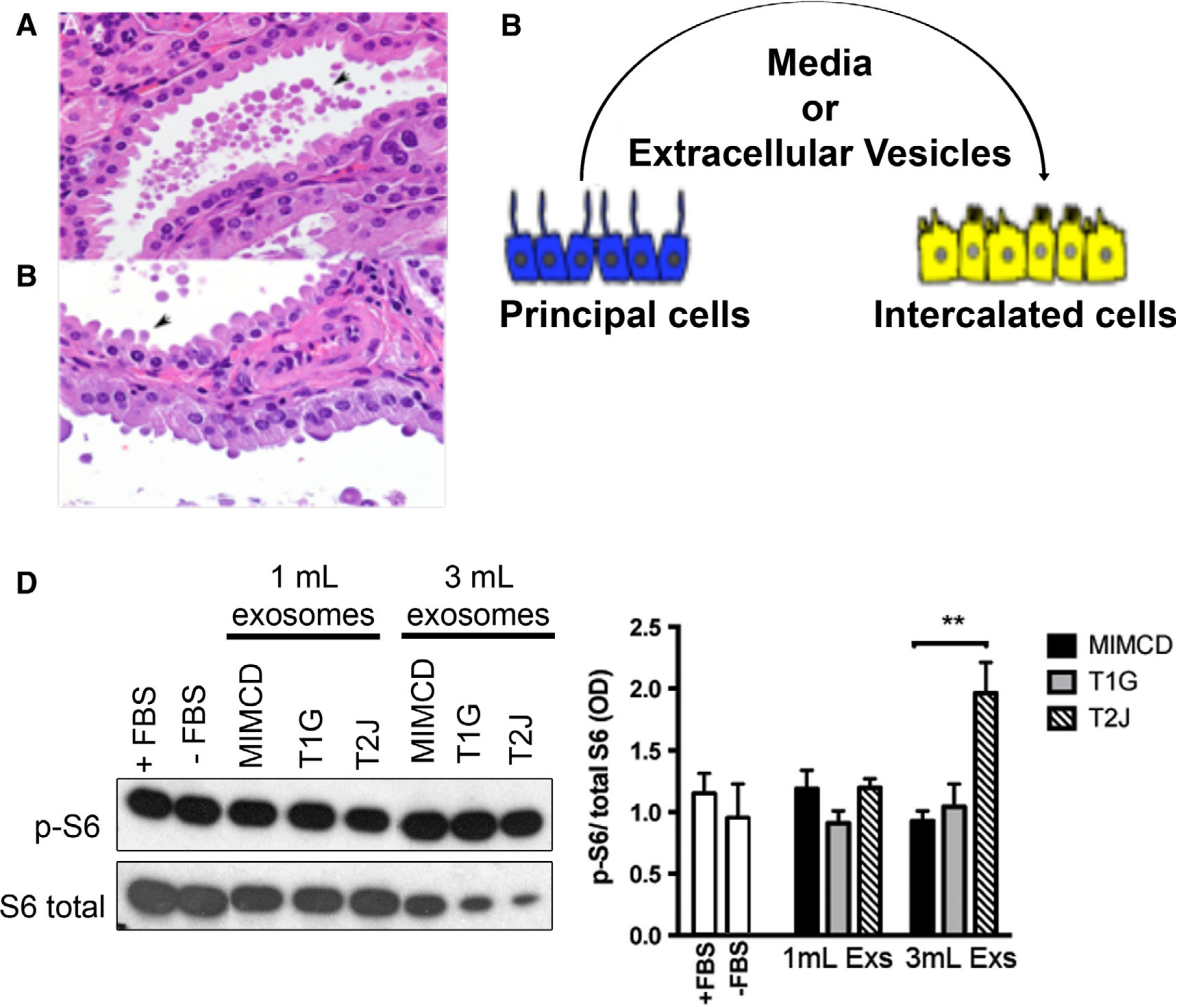
Inner medullary collecting duct (principal) cells produce extracellular vesicle that induce intercalated cell mTORC1 signaling. (A) Microscopic cyst containing extracellular vesicles in lumen (arrow). (B) Extracellular vesicles either budding from or fusing with apical epithelial cell surface (arrow). (C) Diagram of experimental design. Intercalated cells were exposed to principal cell derived conditioned media or isolated extracellular vesicles. (D) Western blot of phospho‐S6 and S6 in cultured intercalated cells. To calibrate experiment, intercalated cells were exposed to FBS or serum starvation (FBS‐), or isolated extracellular vesicles from 1 mL (ECV‐1) or 3 mL (EVC‐3) of the corresponding conditioned media. ** Student *t* test *P* < 0.01.IMCD cells, derived hamartin‐ (T1G and T1H) and tuberin‐knockdown cells (T2H ad T2J). Bar diagram shows optical density of three independent experiments (Mean ± SEM).

To begin to explore possible mechanisms for the phenotypic induction that we described in this manuscript, we tested the probability that extracellular vesicles may be involved. This exploration was based on several reasons. First, such extracellular vesicles are a prominent component of renal pathophysiology (Pomatto et al. 2017), and their release is stimulated by ER stress (Kanemoto et al. 2016) such as that which occurs in cells with a disrupted TSC (Siroky et al. 2012). Second, extracellular vesicle release as well as fusion are known to be increased in an acidic extracellular environment (Parolini et al. 2009; Maas et al. 2017), and the cyst fluid in the *Aqp2CreTsc2* model has a pH = 5.0, likely as a result of increased H^+^‐ATPase expression and activity in the luminal aspect of the cyst. Lastly, increased release of extracellular vesicles has also been shown to occur in states of hypoxia or conditions of increased HIF1α production (King et al. 2012), such as that seen with increased mTORC1 activity driving increased HIF1α (Dodd et al. 2015). In early cysts we were able to identify extracellular vesicles in the cyst lumen (Fig. 9A), and could also detect either vesicle shedding or fusion with the apical epithelial cell (Fig. 9B). To test if these vesicles could mediate the increased mTORC1 activity, we developed a cell culture experiment using IMCD conditioned media by itself, or isolated extracellular vesicles from either one or three milliliters of conditioned media in experiments using intercalated cells (M1 cortical collecting duct cell line) as a target (Fig. 9C). This cell line does not express primary cilia, and morphologically this cell is most compatible with an intercalated cell line (Stoos et al. 1991). We used western blot analysis of phospho‐S6 as a read out of mTORC1 activity.

We used a parental IMCD cells, and isogenic cell lines that were modified to disrupt either *Tsc1* or *Tsc2* using CRISPR/CAS9 technology (Fig. 2A). To examine the possible transfer mTORC1 pathway activity from the IMCD cells to the intercalated cells by extracellular vesicles, we determined the phosphorylation of S6 kinase of M1 cell lysates after 24 h of exposure to isolated extracellular vesicles (Fig. 9D). Comparing the phospho‐S6 band to the S6 band, there is a significant increase in the phospho‐S6/S6 ratio with the higher dose of extracellular vesicles derived from the Tsc2‐deficient IMCD cells (Fig. 9D).

## Discussion

**Figure 10:**
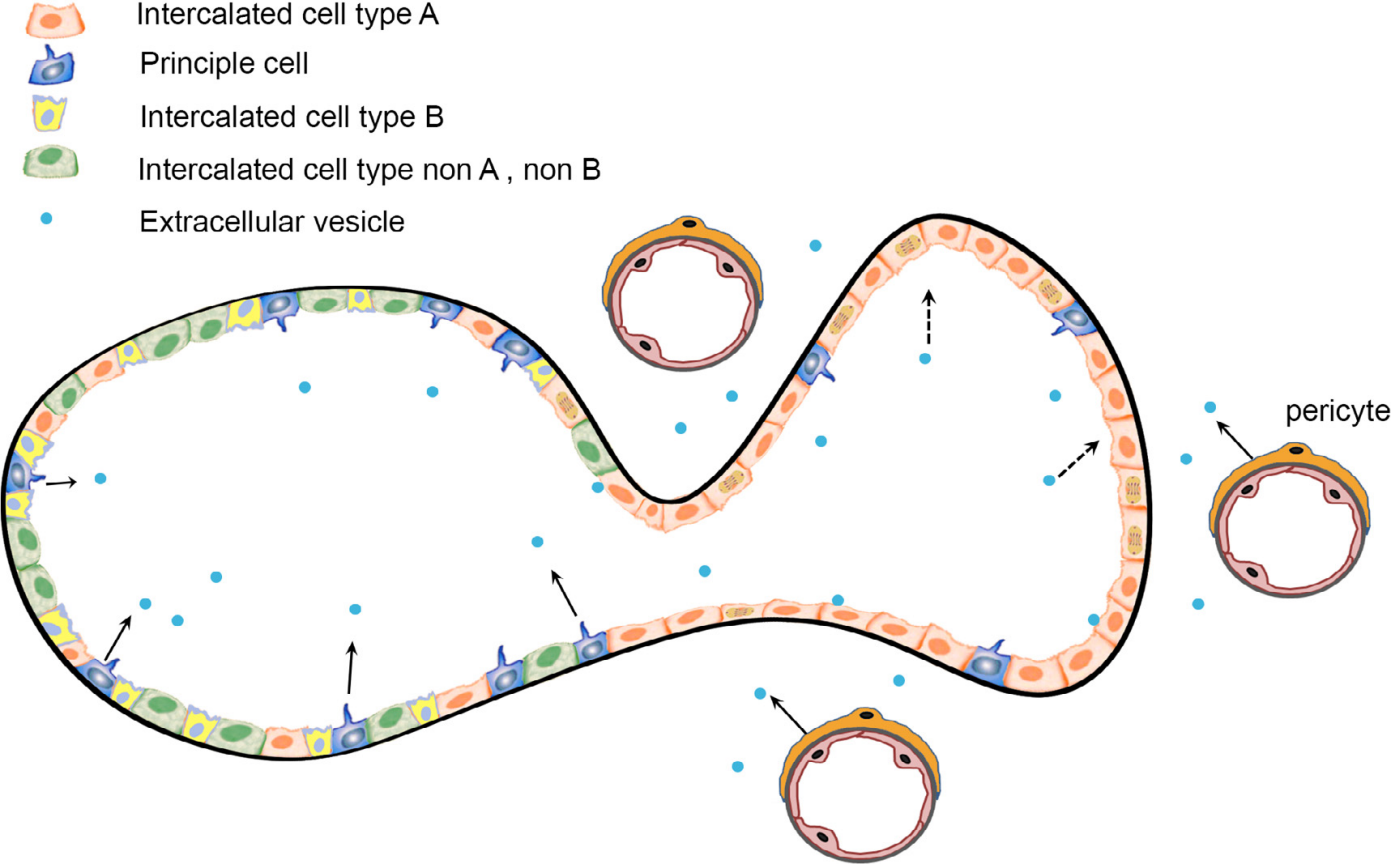
Model of TSC Cystogenesis. Cortical collecting duct contains three types of intercalated cells (see color key) and principal cells that express cilia (blue). Stimulation initiated from either the mutant principal cells or the mutant vascular pericytes (arrows), drive the type A intercalated cells to adopt a mutant phenotype and proliferate (dashed arrows).

We report a new cellular cross‐talk mechanism resulting in tuberous sclerosis renal cystogenesis. A fundamental feature of this mechanism involves a small population of *Tsc*‐mutant renal principal epithelial cells or pericytes that induce or reprogram genetically normal A‐intercalated cells to upregulate their mTORC1 activity, proliferate, and form renal cysts (Fig. 10). Similar microenvironmental effects that increase malignant potential have implicated extracellular vesicles (Zomer et al. 2015). Extracellular vesicles derived from *Tsc1*‐null cells transform the phenotype of neighboring wild‐type cells in vivo such that the wild‐type cells became functionally similar to *Tsc1*‐null cells (Patel et al. 2016), a phenomenon sometimes termed “phenotypic spreading”. Such extracellular vesicles have been demonstrated to affect renal tubular changes in a von Hippel Lindau zebrafish model (van Rooijen et al. 2018). This extracellular vesicle initiating event has appeal because as the cyst develops, the tubular lumen increases in diameter and exponentially reduces the flow, thus increases the time for the extracellular vesicles to interact with intercalated cells. As the cysts enlarge, they detach from the tubule (Grantham et al. 1987), potentially entrapping the extracellular vesicles in the closed cystic space. The intercalated cells with the increased mTORC1 activity likely also participate in the process both in shedding and uptake of the vesicles. This effect also may be mediated through a cellular reprogramming mechanism that induces a sustained change in cellular activity in a paracrine‐like (principal or pericyte to intercalated cell) or autocrine‐like fashion (intercalated to intercalated cells) (Quesenberry et al. 2015). Such a programming effect has been shown in vitro using cells derived from a TSC patient angiofibroma (Julian and Stanford 2017).

Our mouse models and human samples demonstrate a significantly increased preponderance of H^+^‐ATPase positive A‐intercalated cells and disappearance of principal and B‐intercalated cells in the cystic epithelium (Figs. 4 and 6). The vacuolar H^+^‐ATPase is ubiquitously expressed in the membranes of intracellular organelles, including lysosomes, where it plays a crucial role in their acidification. The H^+^‐ATPase is also found in the plasma membranes of certain specialized cell types, including kidney A‐intercalated cells, epididymis, osteoclasts, and certain tumor cells (Blake‐Palmer and Karet 2009; Brown et al. 2009; Gennari 2011; Lee 2012; Breton and Brown 2013; Crambert 2014). H^+^‐ATPase plays an important role in stimulating chloride secretion via apical chloride channels in the collecting duct (Fernandez et al. 1997). These results support the presence of a mechanism that drives the progressive expansion of A‐intercalated cells and the disappearance of principal cells similar to what is described for the differentiation of ionocytes and keratinocyte maintenance in zebra fish embryos (Janicke et al. 2007).

Communication between H^+^‐ATPase and mTORC1 is implicated in reciprocal amplification of their functions. A significant role for H^+^‐ATPase in mTORC1 regulation and translocation to the lysosomal membrane has been demonstrated in several conditions (Zoncu et al. 2011). The H^+^‐ATPase undergoes amino acid‐dependent interactions with the Ragulator complex, which recruits mTORC1 to the lysosomal membrane during amino acid sensing (Kim and Kim 2016). Genetic deletion of structural components of H^+^‐ATPase suppressed amino acid‐induced S6K phosphorylation in *Drosophila* and mammalian cells (Zoncu et al. 2011). Functional inhibition of H^+^‐ATPase activity by chemical inhibitors or by RNAi abrogated mTOR translocation to lysosomes upon amino acid stimulation (Zoncu et al. 2011). These results demonstrate that amino acids activate mTORC1 by stimulating its translocation to the lysosomal membrane where it forms a super‐complex involving the H^+^‐ATPase (Zoncu et al. 2011), suggesting that H^+^‐ATPase is part of the amino acid‐induced signaling pathway that culminates in mTORC1 activation (Pena‐Llopis et al. 2011).

The reciprocal effect of mTORC1 regulating H^+^‐ATPase also has been identified because mTORC1 upregulated the expression of H^+^‐ATPases in immortalized mouse embryo fibroblast (MEF) cells from *Tsc2* ^−/−^ mice (Pena‐Llopis et al. 2011). In addition, mTORC1 facilitates the assembly of V0 and V1 domains of H^+^‐ATPase. These results (Sancak et al. 2010; Pena‐Llopis and Brugarolas 2011; Pena‐Llopis et al. 2011; Zoncu et al. 2011; Kim and Kim 2016) indicate that TSC gene deletion enhances H^+^‐ATPase expression via mTORC1, which, requires H^+^‐ATPase for sustained activity. This amplification loop may play a significant role in the dysregulation of cell growth, expansion of A‐intercalated cells and cystogenesis subsequent to initial signals by TSC mutant cells. Interestingly, our results demonstrate that, despite this new disease mechanism, mTORC1 inhibition can lead to improvement in the patients suffering from TSC renal cystic disease.

TSC cystogenesis has some significant differences with reported pathogenesis in ADPKD, where kidney cysts may originate from multiple nephron segments but predominantly arises from the collecting duct (Harris 2002; Grantham 2015). Fluid accumulation in ADPKD cysts is caused by active chloride secretion via AVP‐stimulated cAMP‐mediated CFTR activation (Sullivan et al. 1998; de Lemos Barbosa et al. 2016). A prostaglandin E2 (PGE2)‐induced chloride secretion mechanism in collecting duct cells involving cAMP‐CFTR‐ and/or calcium‐dependent Cl^−^ channel also has been identified (Rajagopal et al. 2014). Within cysts originating from collecting duct in ADPKD, both intercalated and principal cells are present and principal cells play an important role in fluid secretion (Sullivan et al. 1998; de Lemos Barbosa et al. 2016). Contrary to cysts in ADPKD, our studies using two disparate models demonstrate that cyst epithelia in adult *Tsc1‐ and Tsc2*‐*KO* mice is almost exclusively comprised of A‐intercalated cells, with very few or no principal cells. As such, intracellular cAMP‐activation by AVP may not be a dominant driving force in cyst fluid accumulation and expansion in TSC. Furthermore, whereas our studies clearly demonstrate a uniform and strong expression of apical PRR in the cyst epithelia in *Tsc‐2* KO mice, PKD cysts show a basolateral localization of PRR in principal cells (Saigusa et al. 2015). The cystic mTORC1‐activated ADPKD cell could still utilize the activation loop involving the basolateral H^+^‐ATPase, but the cells involved and H^+^‐ATPase location would be different than that for the TSC cystogenesis.

The mTORC1 activation may involve the cystoprotein polycystin‐1 (PC‐1) in explaining cystogenesis, and may help explain why the *PKD1/TSC2* contiguous gene syndrome has such a severe phenotype. mTORC1 activity negatively regulates the biogenesis of PC‐1 and proper trafficking of the PC‐1/2 complex to cilia (Fig. 7B). While PC‐1 is located on the cilia of principal cells, it is also found on other cell membranes including intercalated cells (Scheffers et al. 2000; Kim et al. 2014), and is strongly expressed on extracellular vesicles (Hogan et al. 2009). Genetic interaction studies have revealed that PC‐1 downregulation by mTORC1 leads to cystogenesis in *Tsc1* mutants (Pema et al. 2016). These findings potentially explain the severe renal manifestations of the *PKD1/TSC2* contiguous gene syndrome. This mechanism also helps explain the Eker rat model described by the Walker laboratory, that developed severe cystic disease and gave rise to the cell line EKT2 (Cai et al. 2003). It is intriguing, given the inputs of von Hippel Lindau protein (Siroky et al. 2009; Elorza et al. 2012) and folliculin (Hasumi et al. 2009) proteins into mTORC1 activity, to posit that these phakomatoses may also arise from a similar mechanism as TSC renal cystic disease.

The disease phenotypic adaption mechanism identified in our animal models complicates the mechanistic understanding of autosomal dominant disease expression. For autosomal dominant polycystic kidney disease, both somatic mutation (second hit”) and haploinsufficiency mechanisms have been put forward. For TSC renal disease, somatic mutation has been favored and has been shown for angiomyolipomata (Henske et al. 1995), but this is not present in the brain tubers or renal cysts (Henske et al. 1996; Bonsib et al. 2016). Human TSC cystic epithelium continues to express the TSC gene products, hamartin and tuberin (Bonsib et al. 2016). In TSC renal cystic disease, both loss of heterozygosity in the inciting cell, and an induced or reprogrammed gain of function in the genetically normal cyst forming epithelium, is involved. The cystic phenotype may depend on the presence of A‐intercalated cells. The *Aqp2CreTsc2* renal medullary collecting duct cells are devoid of cysts despite their lack of tuberin expression. The cystic disease may require the intercalated cell plasticity for disease phenotype manifestation (Roy et al. 2015). This novel mechanism better explains the TSC brain tubers. The genetically abnormal giant cell could use extracellular vesicles to reprogram normal cells to participate in the tuber formation, as supported by detailed genetic analysis of tubers (Crino et al. 2010). It is intriguing that mutant pericytes that likely give rise to the TSC‐associated renal angiomyolipoma (Siroky et al. 2014) could also be involved in TSC renal cystogenesis. Pericytes that have lost TSC gene function may be responsible for altered blood brain barrier function giving rise to the tensor weighted imaging findings in TSC (Jurkiewicz et al. 2007; Arulrajah et al. 2009; Ertan et al. 2010) and may participate in inducing subependymal giant cell astrocytoma formation, a TSC tumor also sometimes identified without loss of heterozygosity (Parry et al. 2000; Bongaarts et al. 2017; Roux et al. 2017), possibly through this extracellular vesicle mechanism. The expansion of A‐intercalated cell as well as potential role for extracellular vesicles in many aspects of TSC renal cystic disease manifestation offers new therapeutic opportunities for intervention in this disease.

## Conflict of Interest

None declared.
